# A Defense Strategy in *Saccharomyces cerevisiae* Mediated by the Golgi Chloride Transporter Gef1p to Cope with Nickel Stress

**DOI:** 10.3390/molecules31193538

**Published:** 2026-10-04

**Authors:** Rongqiu Huang, Xiaoyong Hu, Qianqian Li, Lixuan Zong, Joseph Brake, Chaoyang Luo, Binzhan Wang, Ze Wen, Xiaobin Wu

**Affiliations:** 1 Development Center of Plant Germplasm Resources, College of Life Sciences, Shanghai Normal University, Shanghai 200234, China; 2 Department of Biological Chemistry, University of Michigan, Ann Arbor, MI 48109-0600, USA

**Keywords:** nickel, *GEF1*, *MMT2*, *CUP1*, yeast

## Abstract

Nickel is a widespread environmental pollutant originating from industrial activities, and its pollution problem persists globally. Currently, research on nickel ion metabolism and detoxification mechanisms primarily focuses on prokaryotes, while the molecular mechanisms of nickel detoxification in eukaryotes remain underexplored. In this study, *Saccharomyces cerevisiae* was used as a eukaryotic model to demonstrate that the plasma membrane-localized chloride transporter Gef1p participates in nickel ion metabolism. The *GEF1* knockout strain (*gef1Δ*) showed strong resistance to excess nickel ions, and the content of nickel ions in *gef1Δ* cells was significantly elevated. The results of transcriptomics analysis showed significant upregulation of *MMT2* and *CUP1* in *gef1Δ* cells supplemented with nickel. Both *MMT2* and *CUP1* overexpressed in the *gef1Δ* strain showed a growth advantage on nickel media. Nickel ion content in the mitochondria of cells overexpressing *MMT2* was significantly elevated, and the levels of reactive oxygen species were significantly decreased in strains overexpressing either the *MMT2* or *CUP1* genes. This study reveals that the *GEF1* gene plays an important role in nickel resistance, and that upregulation of *MMT2* and *CUP1* is critical for *gef1Δ* strains to counteract ROS formation and growth defect by nickel ions. The high intracellular accumulation of nickel ions caused by *gef1Δ* during the process of resisting nickel toxicity makes it a strong candidate for ecological remediation of nickel ion pollution.

## 1. Introduction

Nickel is an essential trace element that participates in various physiological activities necessary for plants, animals and microorganisms, and is also a necessary micronutrient for the human body [1]. After being ingested into the human body, nickel resides in various organs such as the brain, liver and heart, where it is believed to play a role in the normal physiological activities of the human body [2]. However, nickel also represents a significant environmental pollutant of concern. As a redox-active metal primarily existing in the +2 oxidation state, nickel exhibits dual characteristics as both an essential element and a toxicant depending on exposure levels. Excess nickel has detrimental effects through its action as a ROS generator. For example, nickel inhibits differentiation of neuronal cells [3], causes premature ovarian failure by inhibiting cell proliferation [4], and causes oxidative damage to the testis, which inhibits sperm formation [5]; long-term exposure to high-nickel environments can also lead to collective epigenetic changes, increasing the risk of cancer [6]. Nickel was shown to inhibit the proliferation of cultured immune cells [7], and reversal of this inhibition is of great interest for the prevention and treatment of nickel-induced cancer. Furthermore, excess nickel ions substitute the essential metal of metalloproteins and result in non-specific binding to catalytic residues of non-metalloenzymes [8]. On the other hand, many microbial enzymes depend on the presence of nickel for their functions, such as urease, methyl coenzyme M reductase, CO-dehydrogenase, nickel superoxide dismutase, glyoxalase, acireductase dioxygenase, lactate racemase, prolyl cis-trans isomerase and [NiFe] hydrogenase [9]. Therefore, organisms must maintain nickel homeostasis to prevent toxicity but allow for its essential functions. However, due to sources such as industrial activities, fossil fuel combustion, smelting, wastewater discharge, electroplating, and vehicle emissions, nickel accumulation in the atmosphere, water bodies, and soil has significantly exceeded the threshold, making nickel pollution a serious threat to ecosystems and human health [10].

A complex two-way interaction exists between microorganisms and environmental nickel: nickel, as an essential trace metal, participates in microbial osmotic regulation and metalloenzyme catalytic activity, while excessive nickel exerts toxic effects by inhibiting soil enzyme activity and altering microbial community structure [11,12,13]. Simultaneously, microorganisms tolerate nickel stress through various mechanisms such as extracellular chelation, intracellular chelation, active efflux, and bioprecipitation [11]. These characteristics make microorganisms a promising candidate for bioremediation of nickel pollution [12]. At present, two homeostasis pathways of nickel have been found in microorganisms. One of the ways for microorganisms to absorb nickel is the nickel-specific ABC transporter found in Escherichia coli, which is composed of five proteins, NikA, B, C, D and E, and relies on ATP hydrolysis to provide energy [13]. Other microorganisms have similar nickel transporters such as NixA, a high-affinity nickel transporter responsible for Ni^2+^ uptake in Helicobacter pylori [14]. The second way for microorganisms to absorb nickel is by facilitated diffusion through nickel-specific permeases or other metal ion transporters [15]. Nickel ions act as essential trace elements for many prokaryotes, enabling a range of reactions involved in energy metabolism. There are few studies on nickel metabolism in eukaryotes such as humans or animals and no specific nickel-containing enzymes have been identified, despite nickel being recognized as an essential trace mineral [16]. Existing studies have pointed out that divalent metal ion transporters, including the NRAMP family, ZIP family, and CDF family, may mediate the transport of nickel ions [17,18,19]. Thus, the mechanism of nickel ion transport in eukaryotes is not clear. Therefore, it is particularly necessary to study the role of nickel in eukaryotes. Some genes are involved in the metabolism of nickel ions. Based on the literature, *MTM1*, *ALR2*, and *ZRT1* in *Saccharomyces cerevisiae*, together with *DMT1* in mammals, have been shown to participate in nickel ion metabolism [8], and more related genes need to be discovered. Previous research in our laboratory found that a *GEF1* knockout strain (*gef1Δ*) showed significant resistance to nickel ions.

The *GEF1* gene belongs to the Guanine nuclear exchange factor (GEF) family and encodes Gef1p, a multifunctional membrane protein located on the Golgi membrane of *Saccharomyces cerevisiae* cells [20]. Previous studies have shown that Gef1p is involved in the regulation of metal ion homeostasis (particularly iron and copper) via its chloride transport activity in the Golgi apparatus [21]. The 13 transmembrane domains encoded by *GEF1* display high homology with chloride ion channel proteins of humans and mammals, demonstrating that Gef1p has ion transport functions [22,23,24]. Studies have also shown that the deletion of *GEF1* leads to increased iron demand and sensitivity to cations in cells [25]. The expression of *GEF1* in HEK293 cells maintains chloride ion channels in an open state, which is essential for the protein’s function [26]. In addition, Gef1p played a key role in the RNA replication of TBSV (tomato bushy stunt virus), and it was verified in *Saccharomyces cerevisiae* that Gef1p knockout inhibited RNA replication of the virus by regulating intracellular copper [21,25]. Therefore, Gef1p is not strictly specific in the regulation of metal ion transport, which provides the possibility for Gef1p to regulate nickel ion transport. At present, many functions of *GEF1* have not been explored, and the metabolic regulation of nickel ions by this gene has not been reported in the literature. Therefore, it is of great significance to investigate the regulatory mechanism between *GEF1* and nickel ions. This work demonstrates, for the first time in *Saccharomyces cerevisiae*, that Gef1p is associated with nickel metabolism, laying the foundation for investigating nickel metabolism in higher eukaryotes.

To investigate the function and regulatory mechanisms of nickel in eukaryotes, the yeast knockout library was used to screen nickel-related genes in *Saccharomyces cerevisiae* with excessive nickel supplementation. In this paper, *GEF1* was found to participate in the metabolic balance of nickel ions, a *GEF1* knockout strain showed resistance to nickel ions, and *gef1Δ* in the presence of nickel caused changes to the expression of several proteins related to metal homeostasis. *MMT2* and *CUP1* were also identified as the key factors for *gef1Δ*-mediated resistance to nickel ions. Mmt2p is located in the mitochondrial membrane, where its function is related to the export of iron ions out of the mitochondria [27], while Cup1p is a metallothionein, which is a metal-binding protein [28]. The results showed that these genes reduce the content of bioactive nickel ions in the cytoplasm, resulting in lower ROS and recovered cell growth, which provides new insights into nickel metabolism in higher eukaryotes and microbial strategies for mitigating nickel contamination.

## 2. Results

### 2.1. Gef1p Is a New Molecular Factor Involved in Nickel Ion Metabolism in Saccharomyces cerevisiae

**Figure 1:**
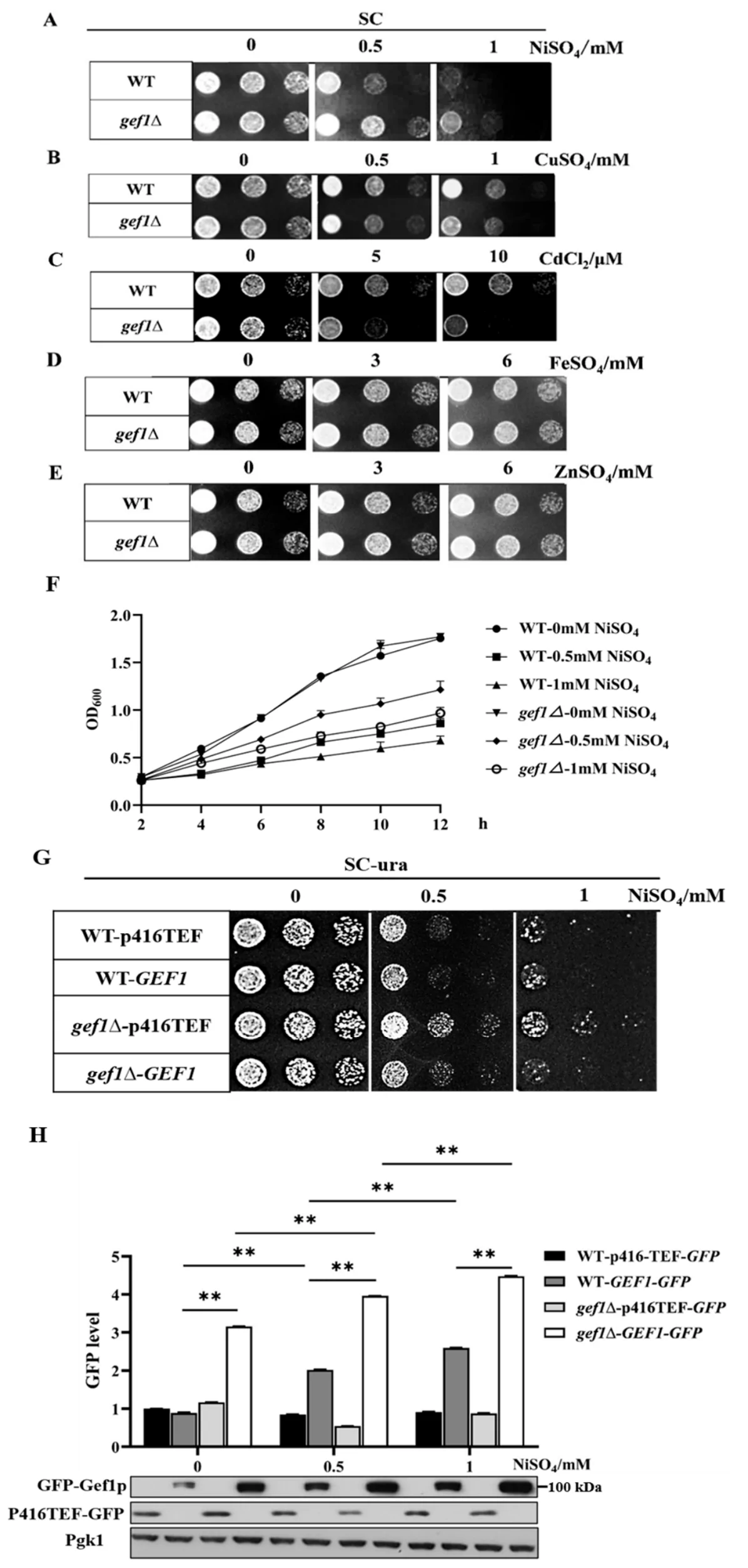
Growth of *gef1Δ* and WT strains on fermentable SC or SC-Ura media with supplementations of indicated metals. WT control and *gef1Δ* strains were cultured with NiSO_4_ (**A**), CuSO_4_ (**B**), CdCl_2_ (**C**), FeSO_4_ (**D**) or ZnSO_4_ (**E**) on SC plates containing glucose to assess growth. (**F**) WT control and *gef1Δ* strains were cultured in SC liquid medium with 0, 0.5 and 1 mM NiSO_4_ and growth curves are shown. (**G**) WT or *gef1Δ* cells were transformed with empty vector (p416TEF) or *GEF1* and grown on SC-Ura medium with or without nickel supplementation. (**H**) Gef1p expression levels in WT and *gef1Δ* overexpressing strains under different concentrations of nickel ion stress (normalization of WB strip grayscale values). There is a one-to-one correspondence between the WB lanes and the bar graphs. All growth tests were carried out with at least four different clones. Significant differences are indicated by asterisks (*), (** *p* < 0.01).

To study the metabolic mechanism of nickel ions in *S. cerevisiae* and identify genes related to nickel ion metabolism, SC solid media containing 0 mM, 0.5 mM, and 1 mM NiSO_4_ were prepared. Then, the growth of gene-deficient strains was observed on the media over three days. *gef1Δ* cells showed enhanced growth versus WT in 0.5–1 mM Ni^2+^-supplemented SC solid medium, suggesting that *gef1Δ* confers nickel ion tolerance (Figure 1A). Next, we confirmed the specificity of the phenotype by repeating the experiment using a dose curve of several other heavy metal ions. *gef1Δ* cells were sensitive to both copper ions and cadmium ions, and their growth trend was noticeably worse than that of WT cells when 0.5 mM copper (Figure 1B) or 5 µM cadmium (Figure 1C) was added. Sensitivity to Cd^2+^ was notably higher than that to Cu^2+^. However, iron ions (Figure 1D) and zinc ions (Figure 1E) had little effect on *gef1Δ* growth. Therefore, the results showed that the ion resistance phenotype of the *gef1Δ* strain was specific to nickel ions. To further verify the experimental results, we prepared a SC liquid culture medium with nickel ion concentrations of 0 mM, 0.5 mM and 1 mM, respectively, and then observed the growth of WT and *gef1Δ* cells. *gef1Δ* exhibited the same nickel ion resistance, as shown in Figure 1A, under nickel ion stress. After adding 0.5 mM and 1 mM nickel ions, the growth trend of *gef1Δ* cells was significantly better than that of WT cells (Figure 1F), consistent with the results of the solid growth experiment, further confirming that *gef1Δ* cells are resistant to nickel ions. Next, the *GEF1* gene was cloned and transformed into WT and *gef1Δ* cells, and their growth trends were observed under the condition of excessive nickel. *gef1Δ* cells still showed resistance to nickel ions after being transformed with the empty plasmid. However, after expressing the *GEF1* gene in *gef1Δ* cells, the cells became sensitive to nickel (Figure 1G). Western blot results demonstrated that overexpression of the *GEF1* gene significantly increased Gef1p levels in cells both before and after the addition of nickel ions. While adding nickel ions elevated the expression of the *GEF1* gene to some degree, it also played a crucial role in stabilizing Gef1p within the cells, especially in the *gef1Δ* strain (Figure 1H). Disparity in inducibility between WT and *gef1Δ* cells contributed to a higher level of Gef1p induction in *gef1Δ* cells (Figure 1H). These results collectively indicate that a regulatory mechanism exists between the *GEF1* gene and nickel ion homeostasis.

### 2.2. GEF1 Deletion Significantly Increases the Nickel Content in Cells Under Condition of Nickel Excess

**Figure 2:**
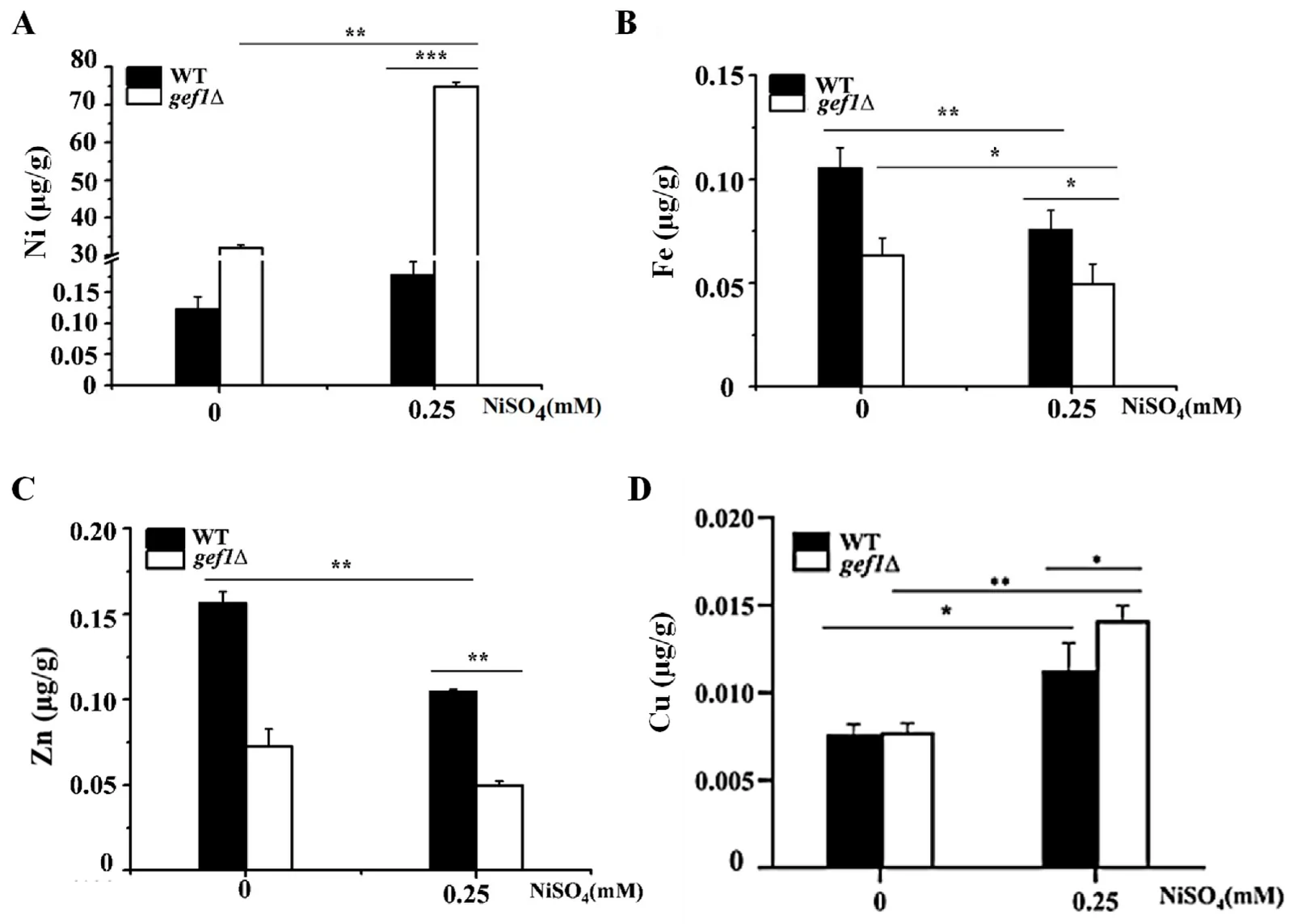
Metal contents in cells of WT control and *gef1Δ* strains. Nickel (**A**), iron (**B**), zinc (**C**) and copper (**D**) in cells were determined by ICP-MS from cells cultured in SC medium with or without NiSO_4_, and then normalized to protein concentration. All metal level analyses were performed with at least four different clones. Average and standard deviation (*n* = 4) are indicated. The asterisk (*) indicates that there are significant differences among different strains with the same treatment (* *p* < 0.05, ** *p* < 0.01, *** *p* < 0.001).

The conjecture proposed that the *gef1Δ* strain’s resistance resulted from lower intracellular nickel levels compared to WT cells. Therefore, ICP-MS was performed to detect the changes in nickel and other divalent metal ions in cells cultured with and without supplemental nickel. The content of nickel in *gef1Δ* cells supplemented with nickel increased relative to WT controls (Figure 2A). However, the contents of iron (Figure 2B) and zinc (Figure 2C) decreased, possibly due to competitive absorption with nickel. However, the copper levels of samples treated with excess nickel ions were significantly higher than those of control samples and the contents of nickel in *gef1Δ* cells were higher than those of WT cells (Figure 2D). Taken together, these data indicate that *GEF1* has a regulatory effect on nickel ions in yeast cells.

### 2.3. MMT2 and CUP1 Are Regulated by GEF1 and Nickel Supplementations

**Figure 3:**
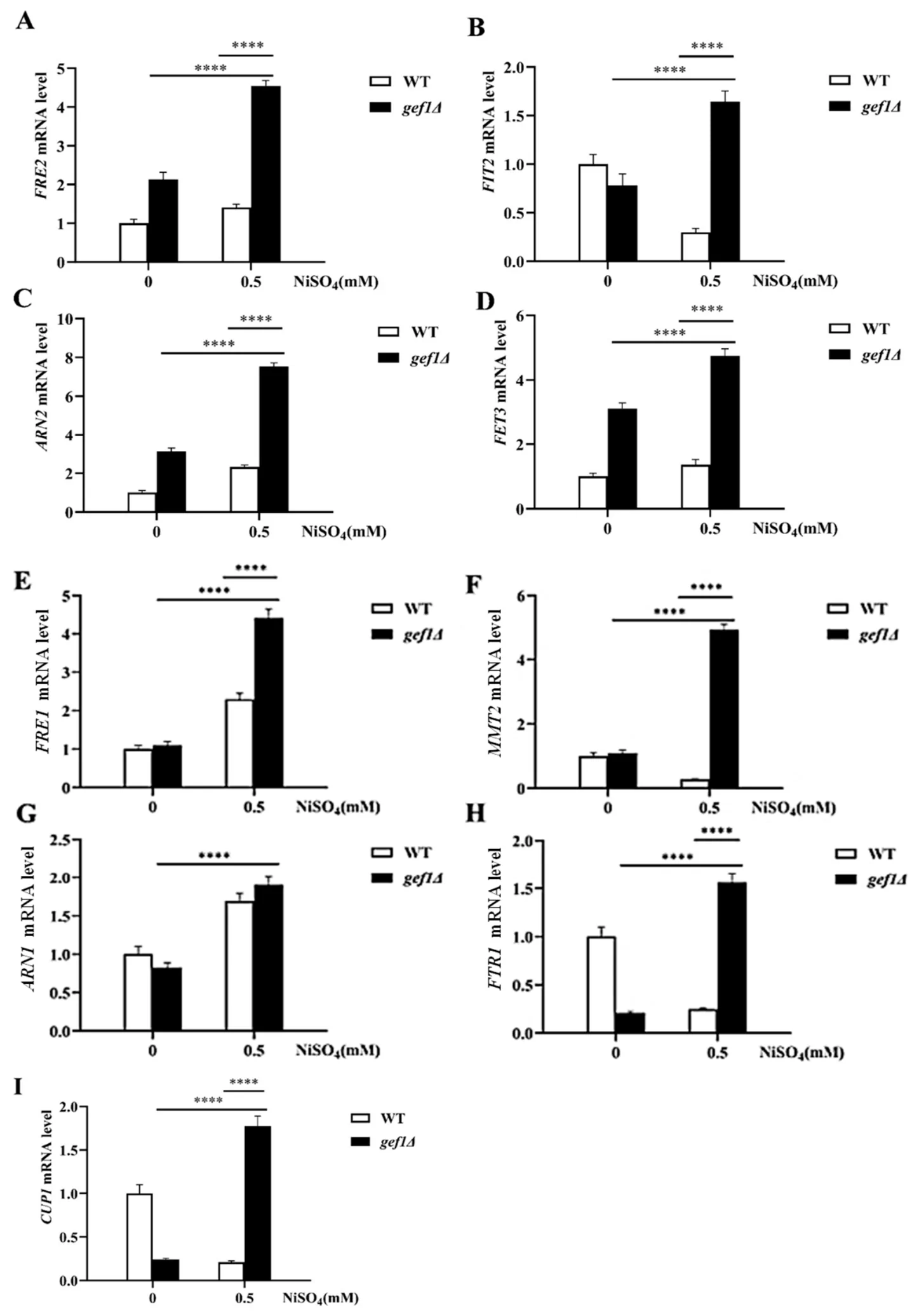
Transcriptomics analysis of WT control and *gef1Δ* strains treated with or without NiSO_4_. Transcriptional fold-change in *FRE2* (**A**), *FIT2* (**B**), *ARN2* (**C**), *FET3* (**D**), *FRE1* (**E**), *MMT2* (**F**), *ARN1* (**G**), *FTR1* (**H**) and *CUP1* (**I**). All treatments were carried out on at least three different clones for Illumina sequencing. Mean and standard deviation values are given. Significant differences are indicated by asterisks (*), (**** *p* < 0.0001).

**Figure 4:**
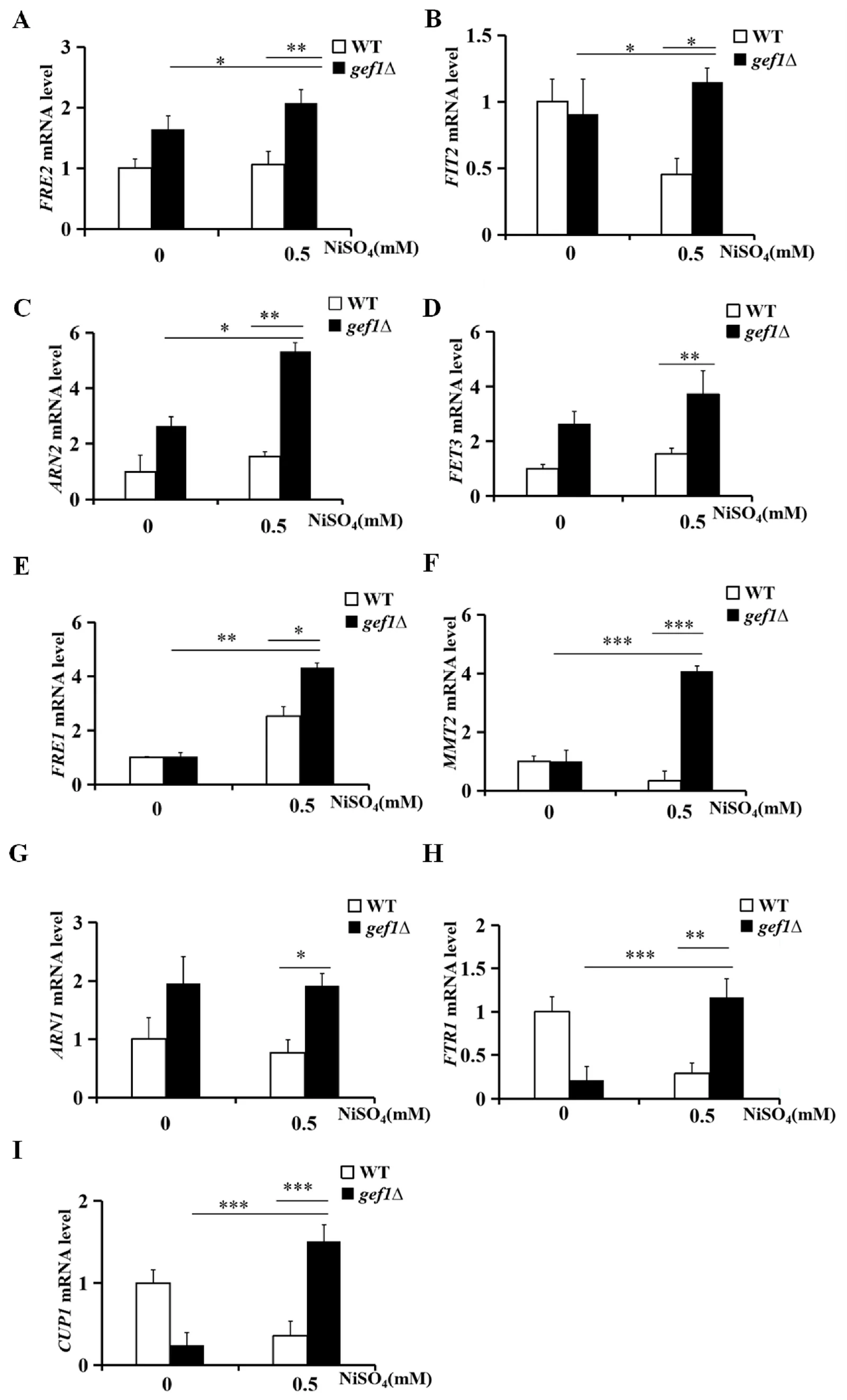
mRNA expression levels of different genes in WT control and *gef1Δ* strains. mRNA expression levels of *FRE2* (**A**), *FIT2* (**B**), *ARN2* (**C**), *FET3* (**D**), *FRE1* (**E**), *MMT2* (**F**), *ARN1* (**G**), *FTR1* (**H**) and *CUP1* (**I**). All treatments were carried out on at least four different clones. Mean and standard deviation values are given. Significant differences are indicated by asterisks (*), (* *p* < 0.05, ** *p* < 0.01, *** *p* < 0.001).

Given that Gef1p is not known to directly affect nickel, we supposed that changes to gene expression in *gef1Δ* cells exposed to nickel were responsible for mediating nickel resistance. Therefore, transcriptome sequencing was used to determine changes in gene expression resulting from supplemented nickel in *gef1Δ* cells. Our previous research found that nickel ions in eukaryotic cells can be transported nonspecifically via other metal ion transport proteins [8]. The related functions of differentially expressed genes were evaluated, and nine Ni-related genes were selected, which are all related to metal ion metabolism. The transcriptional expression levels of the nine genes were then analyzed in WT and *gef1Δ* cells under supplemental nickel ions. Compared with the WT cells, the expression levels of *FRE2* (Ferric REductase), *FIT2* (Facilitator of Iron Transport), *ARN2* (Siderophore iron transporter ARN2), *FET3* (FErrous Transport), *FRE1* (Ferric REductase), *MMT2* (Mitochondrial Metal Transporter), *ARN1* (Siderophore iron transporter ARN1), *FTR1* (Fe TRansporter) and *CUP1* (Cu, copper, CUPrum) all significantly increased (Figure 3). Among these genes, regardless of nickel ion treatment, the transcription levels of *FRE2*, *ARN2*, and *FET3* in *gef1Δ* were significantly higher than in WT cells. This trend was more pronounced under 0.5 mM NiSO_4_ stress (Figure 3A,C,D). Without nickel ion treatment, there were no significant differences in *FIT2* and *MMT2* levels between WT and *gef1Δ* cells (Figure 3B,F). However, under 0.5 mM NiSO_4_ stress, *FIT2* and *MMT2* levels in *gef1Δ* cells were significantly higher than those in WT (Figure 3B,F). The *FTR1* and *CUP1* genes showed a consistent trend with and without nickel ion treatment (Figure 3H,I). Treatment with 0.5 mM NiSO_4_ resulted in significantly higher expression of *FTR1* and *CUP1* in the *gef1Δ* strain than in the WT (Figure 3H,I). However, under nickel ion pressure-free conditions, *FTR1* and *CUP1* in WT and *gef1Δ* exhibited opposite trends (Figure 3H,I). *FRE1* and *ARN1* demonstrated similar trends in transcriptome analysis (Figure 3E,G). Meanwhile, the mRNA expression levels of nine genes were further confirmed by qPCR and the results of *FRE2*, *ARN2*, *FET3*, *FIT2*, *MMT2*, *FTR1*, *CUP1*, *FRE1* were consistent with these of transcriptome sequencing (Figure 4), *ARN1* showed slight differences compared to the transcriptomics results (Figure 4G). qPCR results indicated no significant difference in the transcription levels of the WT and *ARN1* genes before and after nickel ion treatment. Mitochondria play a vital role in cellular defense against external stresses. Since the *MMT2* gene encodes a metal ion transporter located on the mitochondrial membrane, which is closely related to iron transport, and *CUP1* is a type of metallothionein with a high affinity for metal ions, *MMT2* and *CUP1* were prioritized for further research based on the analysis of differential gene transcription levels and gene function.

### 2.4. Mmt2p Affects the Transport of Excess Nickel into Mitochondria and Increases Mitochondrial Sod2p Activity

**Figure 5:**
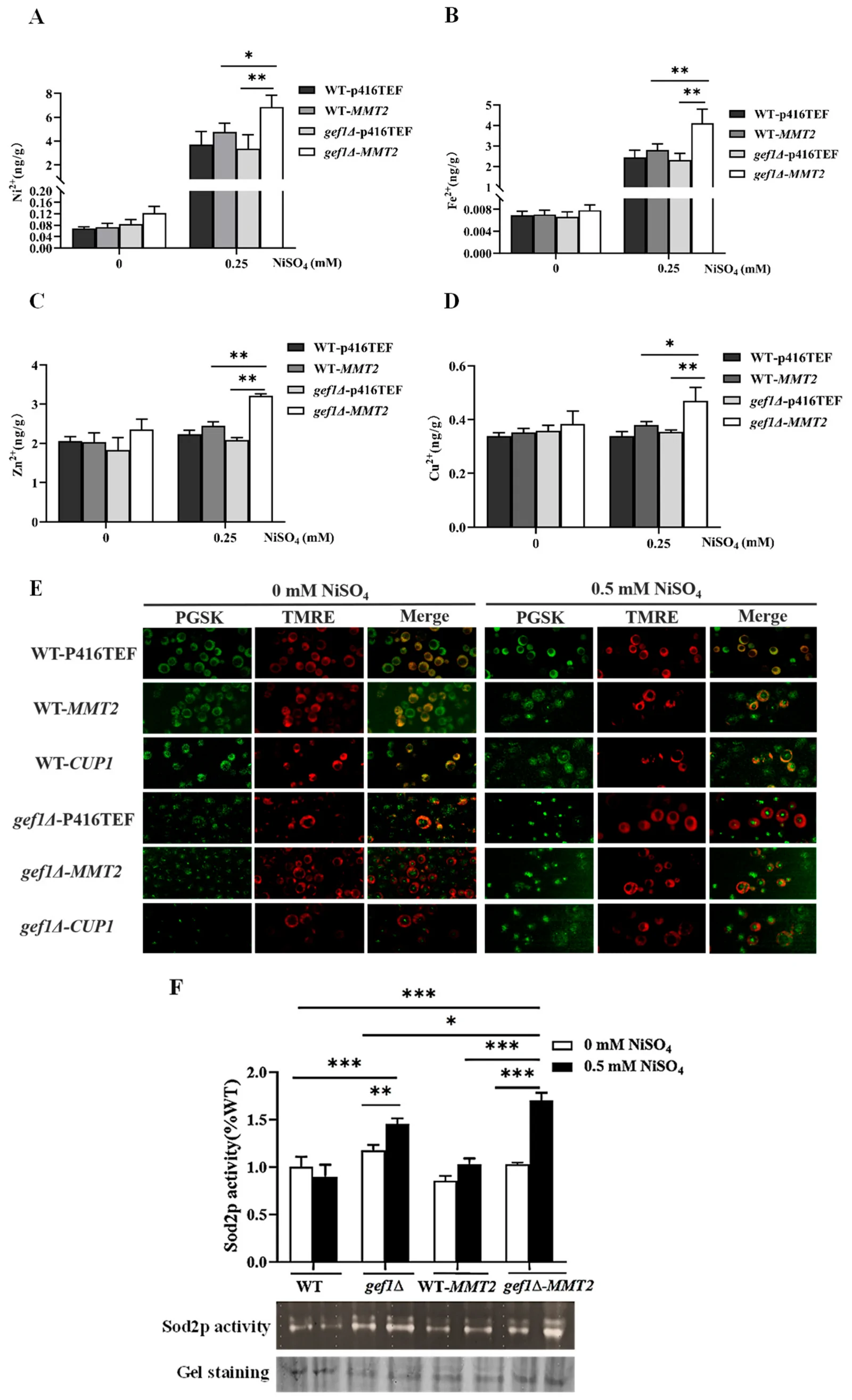
Sod2p activities and heavy metal contents in isolated mitochondria during *MMT2* overexpression in *gef1Δ* strain. Nickel (**A**), iron (**B**), zinc (**C**), and copper (**D**) levels in mitochondria during *MMT2* overexpression were determined by ICP-MS and normalized to mitochondrial protein concentrations. (**E**) Effects of *MMT2* and *CUP1* on intracellular nickel ion distribution in WT and *gef1Δ* cells. The green fluorescent dye is quenched upon binding to nickel ions, whereas the red dye labels mitochondria. (**F**) Sod2p activities of *MMT2* overexpression in WT or *gef1Δ* strains. In-gel assays were conducted with NiSO_4_ treatments in the media for 4 h before collecting cells. All metal level analyses were performed with at least four different clones. Mean ± standard deviation values (n = 4) are indicated. Asterisks (*) indicate significant differences between strains with the same treatment. (* *p* < 0.05, ** *p* < 0.01, *** *p* < 0.001).

Mmt2p is a metal ion transporter localized in mitochondria [29]. It was previously identified to play a role in mitochondrial iron accumulation [29]. Recent studies have found that Mmt2p also functions to export iron ions from the mitochondria into the cytosol [30]. Therefore, Mmt2p may not function as a simple unidirectional transporter in mitochondrial metal ion homeostasis. Previous studies have shown that transferrin and other iron-chelating proteins play a crucial role in nickel ion transport. This may result from the competitive binding and transport of iron and nickel ions by these proteins, or it could be due to the balance of iron ions in cells regulating nickel ions distribution [13,31,32]. Given the function of *MMT2*, we speculated that Mmt2p can transport nickel ions into the mitochondria. To verify this conjecture, we used ICP-MS to detect the content of nickel ions in isolated mitochondria when the *MMT2* gene was overexpressed in WT and *gef1Δ* cells. Compared with the overexpression of an empty plasmid in *gef1Δ* cells treated with nickel ions, the nickel ion content in the mitochondria of the *gef1Δ* cells with *MMT2* gene overexpression increased significantly (Figure 5A). However, this trend was not significant in WT cells. Moreover, the contents of iron, zinc and copper also increased (Figure 5B–D). It can be concluded that when Gef1p is deficient, Mmt2p can transport nickel ions from the cytoplasm to the mitochondria, thus reducing the toxic effects of nickel ions on cells. To further verify this conclusion, we used confocal analysis to detect the distribution of nickel ions in WT and *gef1Δ* cells overexpressing *MMT2* and *CUP1*. The results showed that overexpression of *MMT2* and *CUP1* increased nickel ion accumulation in mitochondria, but not in vacuoles. This trend was more pronounced in *gef1Δ* cells (Figure 5E). This could be one of the reasons *gef1Δ* cells are resistant to nickel ions. Furthermore, our previous study demonstrated that nickel-requiring Sod2p antioxidant enzyme activity is enhanced by the transport of nickel ions into the mitochondria [33]. To prove that *MMT2* overexpression increases Sod2p activity under excess nickel conditions, WT and *gef1Δ* cells expressing the control vector (p416-TEF) or p416-TEF-*MMT2* were cultured with or without NiSO_4_ supplementation to measure Sod2p activity. In-gel oxidase assays indicated that Sod2p activities of *gef1Δ* cells expressing the control vector or *MMT2* were significantly higher than those of WT cells expressing the control vector or *MMT2* under the same Ni treatments (Figure 5F). Therefore, under high-nickel conditions, the increase in Sod2p activity in the *gef1Δ* strain significantly improved the ability of *gef1Δ* to resist oxidative stress. The increase in nickel ions leads to an imbalance in mitochondrial ion homeostasis, and the compensatory increase in iron ions, which causes oxidative stress, can be mitigated by Sod2p, allowing the *gef1Δ* strain to maintain good growth in a high-nickel environment [34].

### 2.5. Overexpression of MMT2/CUP1 Reduces Intracellular ROS Levels

**Figure 6:**
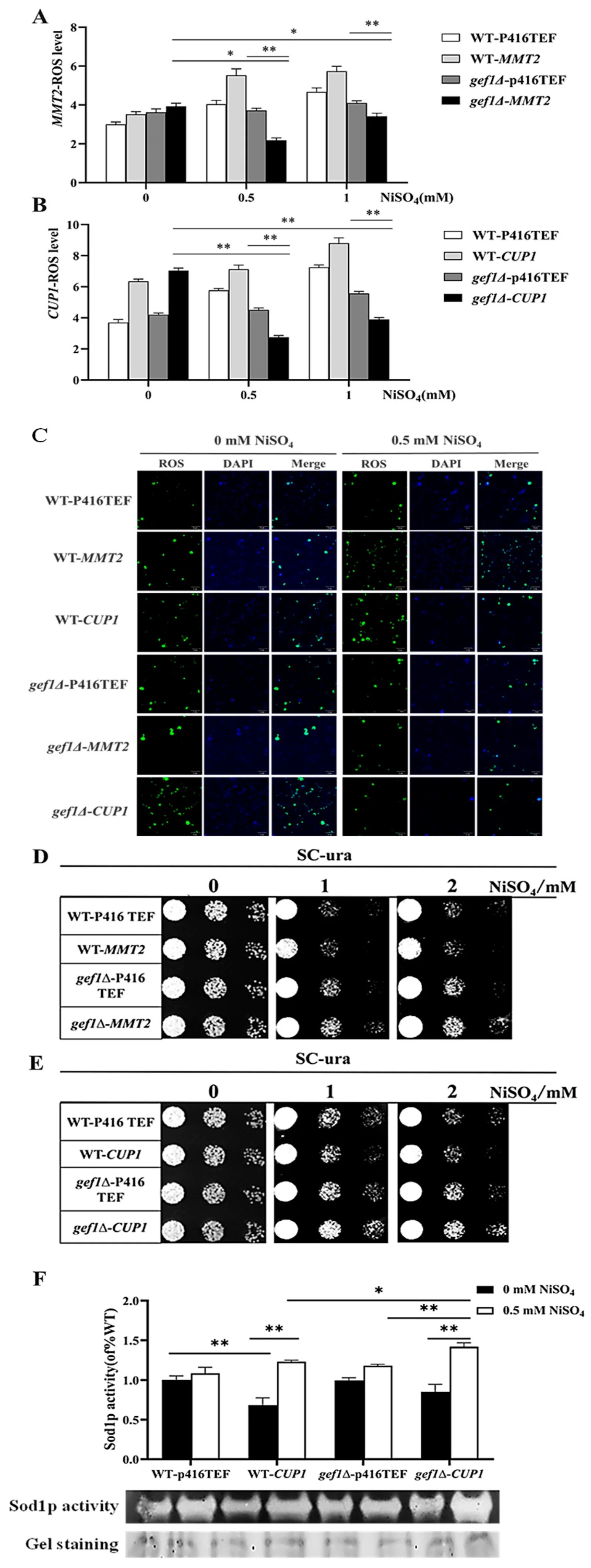
*MMT2*/*CUP1* overexpression reduces intracellular ROS levels. (**A**) Levels of reactive oxygen species produced by *MMT2* when overexpressed in WT and *gef1Δ* strains. (**B**) Levels of reactive oxygen species produced by *CUP1* when overexpressed in WT and *gef1Δ* strains. (**C**) DCFH-DA fluorescence detection by *MMT2* and *CUP1* when overexpressed in WT and *gef1Δ* strains. (**D**) Spot assay of *MMT2* overexpressed in WT and *gef1Δ* strains. (**E**) Spot assay of *CUP1* overexpressed in WT and *gef1Δ* strains. (**F**) Sod1 activities of *CUP1* overexpression in WT or *gef1Δ* strains. All treatments were performed on at least four different cell lines. Means ± standard deviation are given. Significant differences are indicated by an asterisk (*). (* *p* < 0.05, ** *p* < 0.01).

*CUP1* encodes a metallothionein that binds copper and other intracellular metals. Because nickel ions competitively bind with other metal ions, under high-Ni^2+^ conditions, upregulation of *CUP1* in *gef1Δ* cells may promote competitive Ni^2+^ binding, thereby reducing the toxicity of free nickel. Also, *MMT2* was predicted to mitigate ROS production by transporting more nickel ions into the mitochondria to increase Sod2p activity. Therefore, we overexpressed *MMT2* and *CUP1* under high-nickel conditions and found that the ROS levels in *gef1Δ-MMT2* and *gef1Δ-CUP1* strains were significantly reduced compared those in to WT (Figure 6A–C). Additionally, the *gef1Δ-MMT2* and *gef1Δ-CUP1* strains grew better than control cells (Figure 6D,E). These results provided evidence that the upregulation of *MMT2* and *CUP1* by *gef1Δ* + nickel protected cells from nickel-mediated ROS damage. In cells not treated with NiSO_4_, the ROS levels of WT-*CUP1* and *gef1Δ-CUP1* were significantly higher than those of WT-p416TEF and *gef1Δ*-p416TEF. This could be due to an imbalance of other metal ions in WT-*CUP1* cells leading to elevated ROS. Sod1p activity analysis for strains overexpressing *CUP1* found that the Sod1p activity of the WT-*CUP1* strain was significantly lower than that of WT-p416TEF and *gef1Δ*-p416TEF, and the activity of the *gef1Δ*-*CUP1* strain was slightly lower than that of WT-p416TEF and *gef1Δ*-p416TEF in the absence of nickel (Figure 6F). Upon nickel exposure, *CUP1* overexpression significantly enhanced Sod1p activity (Figure 6F). This indicated that, in the absence of nickel ions, the overexpression of *CUP1* likely enhanced the binding capacity for copper ions, resulting in a reduction in intracellular free copper, which affected the activity of Sod1p, and ultimately led to the accumulation of ROS [35]. Similarly, when excess nickel ions are present, nickel ions in the *gef1Δ* strain compete with copper ions to bind to Cup1p, increasing the amount of accessible copper and, to some extent, boosting Sod1p activity, which helps clear ROS in *gef1Δ* cells. However, this pattern is less clear in WT cells, indicating that Gef1p plays a certain regulatory role in Cup1p function.

### 2.6. Nickel Ions Play an Important Role in Regulating mRNA Levels and Protein Expression of Gef1p

**Figure 7:**
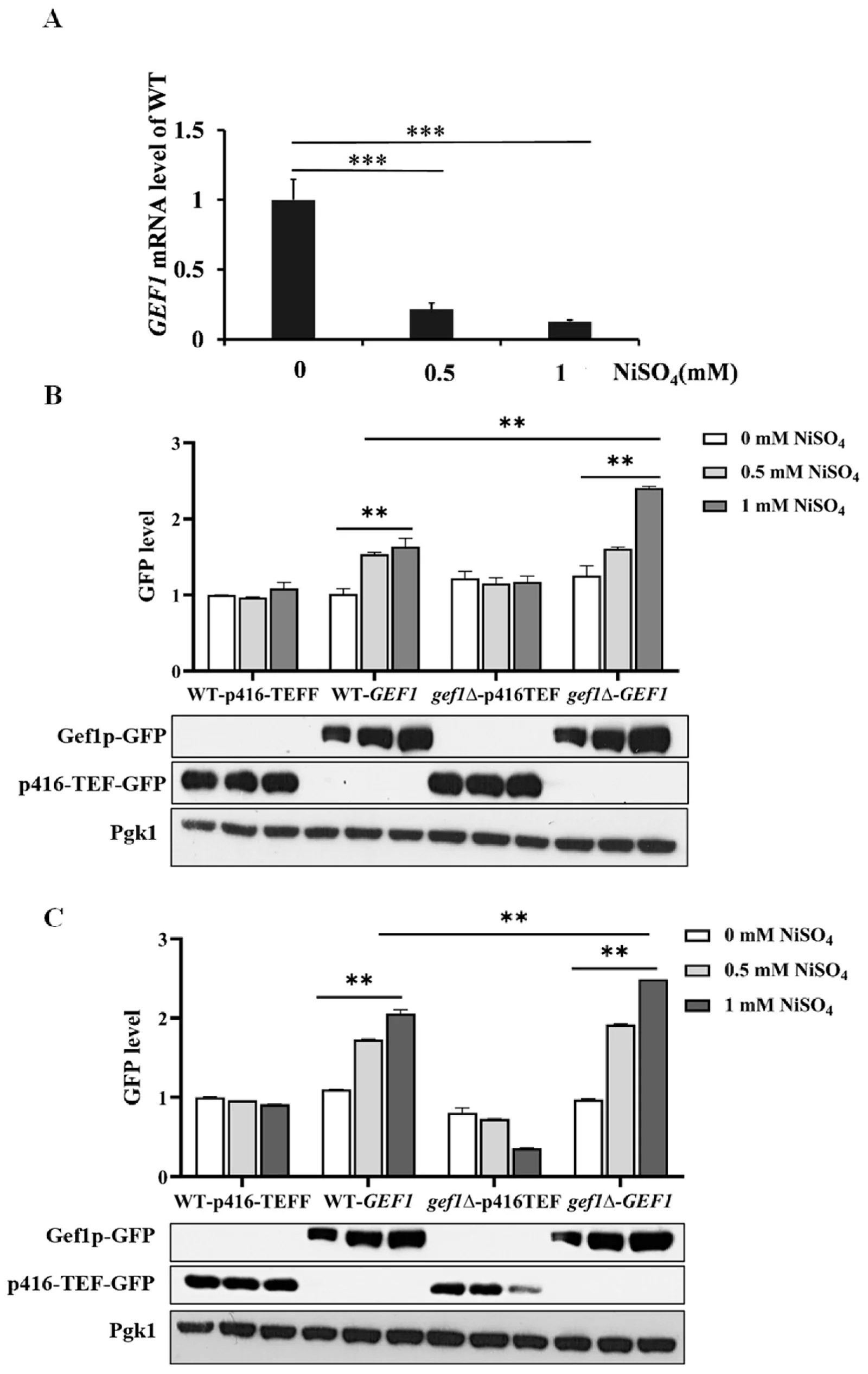
Nickel ions regulate mRNA expression and protein expression levels of *GEF1*. (**A**) mRNA expression levels of *GEF1* in WT strains treated with different concentrations of nickel ions. (**B**) Protein expression levels of p416-TEF-GFP and *GEF1*-GFP in WT cells and *gef1Δ* cells before cycloheximide addition. (**C**) Protein expression levels of p416-TEF-GFP and *GEF1*-GFP in WT cells and *gef1Δ* cells after cycloheximide addition. All treatments were performed on at least three different cell lines. Mean values ± standard deviation are given. Significant differences are indicated by an asterisk (*). (** *p* < 0.01, and *** *p* < 0.001).

*GEF1* directly or indirectly regulates the expression of *MMT2* and *CUP1*, and *gef1Δ* exhibits a decrease in ROS that is dependent on *MMT2* and *CUP1*, yet it is not clear whether the increase in nickel ion content in the WT cells affects the expression of *GEF1*. WT cells were cultured with three concentrations of nickel ions (0 mM, 0.5 mM and 1 mM) in the medium, then the RNA of the cells was extracted, and the mRNA expression levels were analyzed. The mRNA expression level of *GEF1* decreased by more than ten times under excess nickel ion conditions (Figure 7A). Next, three concentrations of nickel ions were added to the SC-Ura medium and WT-p416TEF was used as a control to assess the protein expression of WT-*GEF1* and *gef1∆-GEF1* by Western blot. Before and after adding cycloheximide, both *GEF1*-overexpressing cell lines showed increased Gef1p expression proportional to the concentration of nickel in the medium (Figure 7B,C). Overall, these results revealed that the addition of exogenous nickel ions to the culture medium enhances the protein expression stability of Gef1p, which indirectly indicates that not only can *GEF1* regulate nickel ions, but nickel ions can also regulate *GEF1*.

## 3. Discussion

In this study, the main purpose was to study the function of *GEF1* in the nickel metabolism of *S. cerevisiae*. We found that the *GEF1*-deficient strain showed resistance to toxicity from media containing 0.5 mM and 1 mM nickel ions. On this basis, by evaluating the regulatory mechanism of *GEF1* on nickel ions, this study provides new clues for further elucidating the molecular mechanism of chloride ion channels in the nickel ion toxicity response.

**Figure 8:**
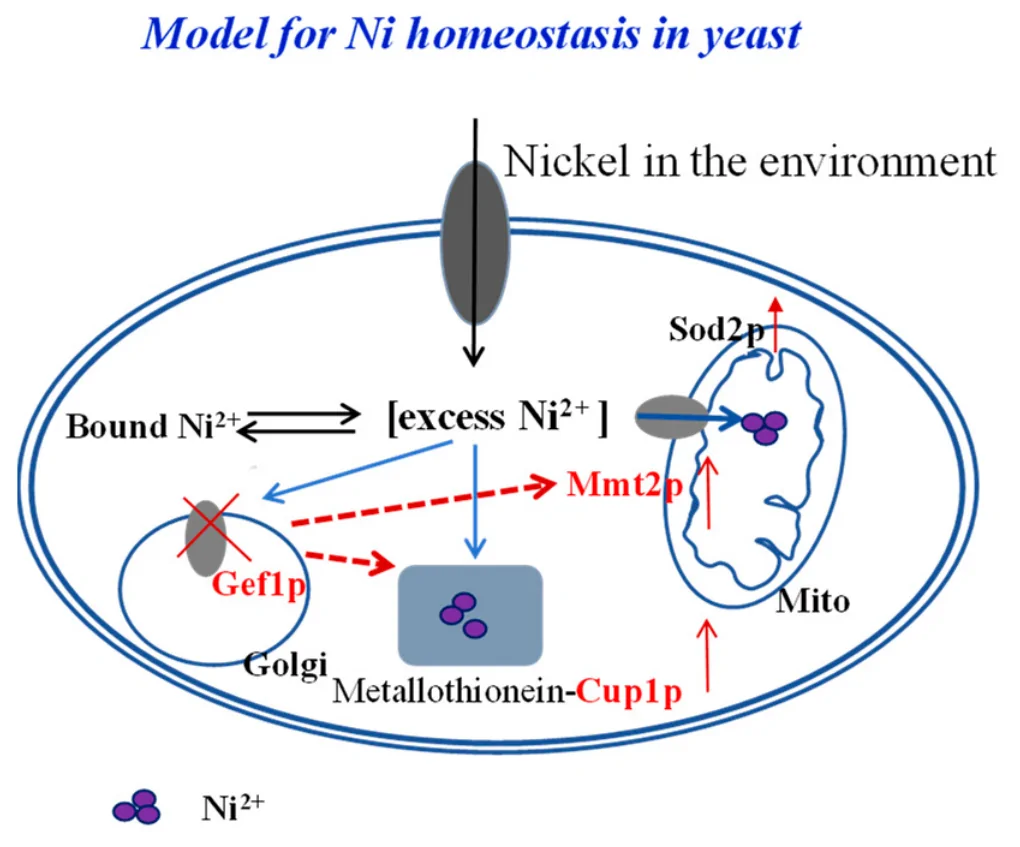
The regulatory mechanism of nickel ions by the *GEF1* gene: In *gef1Δ* cells, excess nickel ions bind to free nickel through Cup1p and are transported into the mitochondria through Mmt2p, thereby increasing Sod2p activity to reduce ROS levels and thus enabling cells to resist damage caused by nickel ion stress. Solid arrows represent substance transport or flow, whereas dotted arrows indicate functional regulation or indirect interactions.

We proposed a model for the regulation of Ni distribution by Mmt2p and reduction in ROS levels by *MMT2/CUP1* overexpression (Figure 8). According to transcriptomics, *GEF1* can maintain intracellular nickel ion levels in a stable range by regulating gene expression. The regulation of nine genes identified through transcriptomics was verified by qPCR, with a focus on two highly upregulated genes with potential roles in nickel metabolism: *MMT2* and *CUP1.* Mmt2p may not function as a simple unidirectional transporter in mitochondrial metal ion homeostasis. Based on previous results from our laboratory, we concluded that most proteins that transport iron ions can also play an important role in nickel ion transport, so Mmt2p is likely to regulate nickel ion transport from the cytosol to the mitochondria [33]. In addition to regulating overall cellular toxicity, elevated mitochondrial nickel levels also enhance the activity of Sod2p, which plays a critical role in counteracting oxidative stress [33]. *CUP1* encodes a metallothionein, which is a metal-binding protein. When *GEF1* exists, the increase in Ni^2+^ content and reduction in available copper within cells results in a greater rise in ROS compared to *gef1Δ* (Figure 6A–C). The accumulation of ROS inhibits the transcription of iron-regulating factors such as Aft1/2, leading to an imbalance in intracellular iron homeostasis, which may indirectly inhibit the transcription of *MMT2* [36]. Additionally, ROS can impact the function of *CUP1* transcription factors (*ACE1/MAC1*), oxidize Cys residues, and contribute to the downregulation of *CUP1* [37]. The increased transcription of *MMT2* and *CUP1* in *gef1Δ* cells under nickel ion treatment was likely to lead to the accumulation of nickel ions in cells and mitochondria (Figure 3F,I and Figure 4F,I). Therefore, we used inductively coupled plasma mass spectrometry (ICP-MS) and solid-state growth experiments in cells overexpressing *MMT2* and *CUP1* to show that Mmt2p promotes the transport of nickel from the cytosol to the mitochondria, and both *MMT2* and *CUP1* restored growth in nickel-supplemented media. Furthermore, the significant reduction in ROS levels and the growth advantage of *gef1∆-MMT2* and *gef1∆-CUP1* compared to WT-*MMT2* and WT-*CUP1* demonstrated that *MMT2* and *CUP1* depend on *GEF1* for nickel resistance (Figure 6A–E). These data suggest that the *gef1Δ* strain was resistant to nickel ions through the upregulation of *MMT2* and *CUP1*, which act to reduce intracellular, redox-active nickel ions by sequestration in the mitochondria and cytosol, respectively. The improvement of Gef1p-dependent nickel resistance is achieved by regulating the cell’s free radical scavenging ability. We also cannot rule out the possibility that the vacuole may play a role in nickel sequestration similar to metal ion transporters in yeast that enable vacuole-mediated metal mobilization and storage [38].

Finally, we identified a reciprocal relationship between *GEF1* and nickel whereby, just as *GEF1* can regulate the metabolism of nickel ions, the nickel ions can also affect the expression of *GEF1*. Real-time fluorescence quantitative PCR experiments showed that nickel ions reduced the mRNA expression level of *GEF1*. The results of Western blot showed that nickel ions could stabilize the expression of Gef1p. A possible explanation for this regulation is that excess nickel in the media impairs the uptake of iron and other metals through competitive inhibition (supported by Figure 2). Therefore, stabilization of Gef1p, a chloride channel implicated in iron and copper homeostasis [39], may be a compensatory mechanism to maintain proper micronutrient balance. To summarize, this work demonstrated new roles for *GEF1* in the absorption, utilization, and storage of nickel ions in cells, providing new insight into studying diseases related to nickel ion metabolism. This will also contribute to a broader understanding of the toxicological effects of nickel as an environmental pollutant.

To further explore the relationship between *GEF1* and nickel ions, it is necessary to identify the mechanism of how nickel ions reduce the mRNA level of *GEF1* and enhance stability of the gef1p protein. A possibility is that nickel ions may directly interact with transcription factors to affect *GEF1* expression in a manner similar to NikR in microbes [40]. Nickel ions may also directly interact with gef1p protein to affect its stability. For example, nickel binding to HpNikR was shown to affect its conformation and stability and nickel dramatically increased the thermostability of UreE [41,42]. Additionally, it is necessary to explore how *GEF1* regulates the transcription levels of *MMT2* and *CUP1* including the extent to which nickel and other metal ions are implicated. Furthermore, it is unknown whether higher eukaryotes or humans have processes analogous to the relationship between *GEF1* and nickel shown in *S. cerevisiae*. Previous studies have demonstrated that human ClC1 and ClC2 can complement the *gef1Δ* phenotype, indicating functional conservation of Gef1p homologs in humans [43]. Consequently, establishing functional equivalence of Gef1p in mammalian cells would be of great significance for elucidating nickel metabolism in higher eukaryotes. Our work hopes to provide a basis for further study of nickel metabolism in eukaryotes which is greatly lacking despite being considered an essential trace mineral.

## 4. Materials and Methods

### 4.1. Yeast Strains, Culture Media, and Growth Assays

A haploid control yeast *S. cerevisiae* strain, BY4741, and the *gef1Δ* strain (BY4741, 00-13-70-F-12) were purchased from Open Biosystems (Huntsville, AL, USA) [44]. Yeast strains were grown in synthetic complete media (SC: mixture of 24 amino acids, yeast nitrogen base) lacking uracil (SC-Ura) for plasmid selection as indicated in the figure legends [45]. Further, 1.5% agar was supplemented into the liquid media for solid medium plates. Yeast strains were cultured at 30 °C.

Yeast cell growth assays were based on our previous methods [46]. Wild-type (WT) or knockout cells transformed with either empty vector, *MMT2* or *CUP1* plasmids were grown overnight in SC-Ura media, re-inoculated (OD_600_ = 0.2) into fresh media, and grown to the mid-log phase (OD_600_ = 0.8–1.0). Cells were spotted on SC media supplemented with the indicated metals. Each treatment was repeated more than 3 times with 3 different colonies.

### 4.2. Plasmid Manipulation

The *GEF1*, *MMT2* and *CUP1* genes amplified by PCR were integrated into Bam HI and XhoI sites in the p416-TEF vector. The GFP (Green Fluorescent Protein) epitope tag was integrated at the C-terminus of Gef1p via a NotI restriction enzyme site before the stop codon was produced in the PCR primer. Conventional molecular biology methods followed previously established technologies [46]. The target genes involved in the plasmid construction process were sequenced and verified. The lithium acetate method was used for yeast transformations [47].

### 4.3. Yeast Transcriptomic Sequencing

WT and *gef1Δ* cells were cultured overnight in SC media, re-inoculated (OD_600_ = 0.2) into new SC media, and grown to the mid-log phase (OD_600_ = 0.8–1.0). Cells were collected and sent to Majorbio Company (Shanghai, China) for Illumina sequencing. Based on differentially expressed gene data obtained from the transcriptome, a graph is plotted based on the level of gene transcriptional differences.

### 4.4. Western Blot Analysis

WT and *gef1Δ* strains expressing the control vector (p416-TEF) or *GEF1*-GFP were grown in SC-Ura media starting at OD_600_ 0.2 for 4 h. Subsequently, the exponentially growing cells were treated with/without cycloheximide (100 μg/mL) for 30 min and then collected for protein stability determination. Total protein was prepared via glass bead disruption in phosphate-buffered saline (PBS) containing 1% Triton X-100 and protease inhibitor complex (Roche Applied Science). The BCA kit (Pierce) was used for protein concentration measurements following the manufacturer’s instructions. Total protein was denatured in SDS buffer containing 100 mM DTT (dithiothreitol) for 15 min at 60 °C and subjected to SDS-PAGE. Gef1p-GFP expression was tested with anti-GFP primary antibody (Rockland) and anti-rabbit IgG secondary antibody (Santa Cruz). Anti-Pgk1 primary antibody (Abcam) was used to test Pgk1 expression level as a control. The WB results were repeated three times. The results were quantitatively analyzed using Image J (1.53K), and the quantitative analysis data were analyzed and plotted using Prism.

### 4.5. Metal Measurement

The contents of metals were measured by inductively coupled plasma mass spectrometry using the method described previously [48]. Data were normalized according to protein concentration.

Intracellular metal ion distribution was detected using the Phen Green SK diacetate (5/6-mixture) probe (Purchased from MCE). Based on the quenching property of the Phen Green SK diacetate (5/6-mixture) probe against nickel ions, strains WT-P416TEF, WT-*MMT2*, WT-*CUP1*, *gef1Δ*-P416TEF, *gef1Δ-MMT2*, and *gef1Δ-CUP1* were cultured overnight in 3 mL of bacterial solution, synchronized to OD_600_ = 0.2, and cultured at 190 rpm and 30 °C for 4–6 h. One OD unit of yeast cells was cultured in 100 mM AcLi at 30 °C for 1 h, followed by treatment with 20 µm Phen Green SK diacetate (5/6-mixture) at 30 °C for 1 h. Cells were washed twice with 1 mL of PBS (pH = 7.4), resuspended in 500 µL of PBS, and observed under confocal microscopy at an excitation wavelength of 507 nm and an emission wavelength of 532 nm.

### 4.6. Quantitative PCR

WT and *gef1Δ* strains were grown in SC media overnight. Cells were then re-inoculated (OD_600_=0.2) into new media and cultured for 4 h with NiSO_4_ (0, 0.5, 1 mM). Total RNA was used to synthesize cDNA followed by quantitative PCR (qTOWER, 3.0 G) with specific primer sets for *MMT2* (F: 5′-AGC AGCGGG TTCTATCTTGG-3′; R:5′-ACCAACGCTACCAACGATGT-3′), *CUP1* (F: 5′-GCCAATGT GGTAGCTGCAAA-3′; R:5′-TCAGACTTGTTACCGCAGGG-3′), *FRE2* (F: 5′-CCTGTCAGGAGCAAGAGCAT-3′; R:5′-CCTCGTGCAGGCATTAGTGA-3′), *FIT2* (F: 5′-GACACCGCTGACCCTATCAT-3′; R:5′-GGTCCTTCGGTGACAACCTT-3′), *ARN2* (F: 5′-GTGGGCCCCATTGGGAATTA-3′; R:5′-CGCTCTCATCTACCGCAACT-3′), *FET3* (F: 5′-AGGTGACCGTGTGCAGATTT-3′; R:5′-CCATAGAGGCGGTTCCGTTT-3′), *FRE1* (F: 5′-CCCAAGCTGCGCTATACCAA-3′; R:5′-AGGCACATGCTGTCACTGAA-3′), *ARN1* (F: 5′-TGGGTGATGTTGCTCTAGCC-3′; R:5′-AAGGCATTCCTTTGTGGGGT-3′), *FTR1* (F: 5′-AACAGGCAATCGGGGAACAT-3′; R:5′-TGAACCCGAGTAACACACCG-3′) and β-actin (F: 5′-A AATGCAAACCGCTGCTCA-3′; R:5′-TACCGGCAGATTCCAA ACCC-3′). The qPCR results were analyzed using a relative quantification method, and plotted using Prism software.

### 4.7. Measurement of Intracellular ROS Level

Intracellular ROS levels were determined by a nitroblue tetrazolium (NBT) assay with some modifications [49]. Briefly, WT and *gef1Δ* cells were cultured to the mid-log phase with indicated NiSO_4_ treatments. Cells were incubated for 90 min in PBS containing 0.2% NBT before being collected. ROS reduced NBT to a dark blue insoluble form called formazan. The mixtures were centrifuged at 5000 rpm for 2 min at 4 °C. The supernatant was removed and the formazan was dissolved in 300 µL of 50% acetic acid by sonication (3 pulses of 6 s). The samples were then centrifuged and the absorbance of the supernatant was measured at 560 nm by a spectrometer.

Intracellular ROS levels were verified using the DCFH-DA fluorescent probe (Beyotime, China). WT and *gef1Δ* cells were cultured to the logarithmic growth phase in the presence of NiSO_4_. One OD unit of cells was then treated with 100 µL of 0.1 mM AcLi at 30 °C and 190 rpm for 1 h, followed by incubation with the DCFH-DA probe for 1 h. Fluorescence was observed using an Olympus FV3000 Confocal.

### 4.8. Yeast Mitochondria Isolation

The *gef1Δ* cells expressing the empty vector (p416-TEF) or *MMT2* were grown in SC-Ura media to the mid-log phase with the indicated treatments. Yeast mitochondrial isolation was based on our previously established method [50].

### 4.9. Superoxide Dismutase 2 (Sod2p) and Superoxide Dismutase 1 (Sod1p) Assay

WT and *gef1Δ* strains expressing the control vector (p416-TEF) or p416-TEF-*MMT2* were grown in SC-Ura media with or without 0.5 mM NiSO_4_ for 4 h. Total protein extracts were prepared and subjected to Sod1p and Sod2p activity measurement based on the previous method [46]. The grayscale values of the gel bands were quantitatively analyzed using Image J.

### 4.10. Statistical Analysis

Image quantification was determined by Image J software (http://rsbweb.nih.gov/ij/, accessed on 20 August 2026). Descriptive analyses are presented as the means ± S.D., and statistical comparisons of control and experimental groups were performed using One-way ANOVA and Student’s *t*-test. *p* < 0.05 was considered to be significant.

## 5. Conclusions

In all, for the first time, our study clarified a new function of *GEF1* associated with nickel resistance. This provides a theoretical basis for understanding the response mechanism of excessive nickel ions to cellular stress. It is hoped that industrial yeast strains with high nickel tolerance can be constructed by using the function of the *GEF1* gene in the future, thus making certain contributions to industrial development. The results also provide potential application value for bioremediation technologies related to heavy metal pollution.

## Data Availability

Data are contained within the article.
